# Injury to Cone Synapses by Retinal Detachment: Differences from Rod Synapses and Protection by ROCK Inhibition

**DOI:** 10.3390/cells12111485

**Published:** 2023-05-27

**Authors:** Ellen Townes-Anderson, Éva Halász, Ilene Sugino, Amy L. Davidow, Laura J. Frishman, Luke Fritzky, Fawad A. K. Yousufzai, Marco Zarbin

**Affiliations:** 1 Department of Pharmacology, Physiology and Neuroscience, Rutgers New Jersey Medical School, 185 South Orange Avenue, Newark, NJ 07103, USA; eh394@njms.rutgers.edu; 2 Institute of Ophthalmology and Visual Science, Rutgers New Jersey Medical School, 90 Bergen Street, Newark, NJ 07103, USA; suginoik@kajohnson.com (I.S.); zarbin@earthlink.net (M.Z.); 3 Department of Biostatistics, New York University School of Global Public Health, 708 Broadway, New York, NY 10003, USA; davida02@nyu.edu; 4 Department of Vision Sciences, College of Optometry, University of Houston, Martin Luther King Blvd, Houston, TX 77204, USA; lfrishma@central.uh.edu; 5 Cellular Imaging and Histology Core, Rutgers New Jersey Medical School, 205 South Orange Avenue, Newark, NJ 07103, USA; fritzklf@njms.rutgers.edu (L.F.); fawad.y@rutgers.edu (F.A.K.Y.)

**Keywords:** cone cell, photoreceptor, synapse, retinal detachment, Rho kinase, synaptic ribbons, pedicle, spherule, STED microscopy, pig

## Abstract

Attachment of a detached retina does not always restore vision to pre-injury levels, even if the attachment is anatomically successful. The problem is due in part to long-term damage to photoreceptor synapses. Previously, we reported on damage to rod synapses and synaptic protection using a Rho kinase (ROCK) inhibitor (AR13503) after retinal detachment (RD). This report documents the effects of detachment, reattachment, and protection by ROCK inhibition on cone synapses. Conventional confocal and stimulated emission depletion (STED) microscopy were used for morphological assessment and electroretinograms for functional analysis of an adult pig model of RD. RDs were examined 2 and 4 h after injury or two days later when spontaneous reattachment had occurred. Cone pedicles respond differently than rod spherules. They lose their synaptic ribbons, reduce invaginations, and change their shape. ROCK inhibition protects against these structural abnormalities whether the inhibitor is applied immediately or 2 h after the RD. Functional restoration of the photopic b-wave, indicating cone-bipolar neurotransmission, is also improved with ROCK inhibition. Successful protection of both rod and cone synapses with AR13503 suggests this drug will (1) be a useful adjunct to subretinal administration of gene or stem cell therapies and (2) improve recovery of the injured retina when treatment is delayed.

## 1. Introduction

Rod photoreceptors evolved from cone photoreceptors [1,2] to give us the capability to see over a wide range of luminosity with sensitivity to low light provided by rod cells and constant activity at ambient light levels provided by cone cells. The evolutionary link between rod and cone cells indicates numerous similarities. There are, however, also many differences in anatomy and physiology and, at the molecular level, in mechanisms such as light transduction and synaptic transmission. It is no surprise, therefore, that the responses of cone and rod cells to mechanical injury and retinal detachment display many differences. Fisher and colleagues [3,4,5,6] were the first to describe some of these differences concerning the synaptopathy of retinal injury. Retinal detachment results in the retraction of rod spherules from their bipolar and horizontal cell partners that, in many cases, do not repair even after long-term reattachment. In contrast, cone synapses do not retract in response to detachment, although their pedicles change shape, invaginations flatten, and ribbons can disappear. Data about cone synapses, however, remain limited in part because the synaptic cone changes are arguably more subtle and because cone markers disappear with increased time after detachment [7]. 

Because of the importance of the cone pathway to visual acuity and color vision, we undertook to expand the knowledge of cone pedicle structure after injury. We used quantitative analysis of confocal images and the new microscopic technique, stimulated emission depletion (STED), applied to a porcine model of retinal detachment. Moreover, because we reported that rod synaptic change occurs within hours of the injury [8], we have examined early time points (2–4 h) after detachment, times for which there is presently no information, as well as times after spontaneous retinal reattachment.

We have previously reported that the rapid changes in rod spherules, i.e., retraction, can be significantly reduced by the use of inhibitors to the RhoA pathway [8,9,10,11,12,13]. RhoA is a key signaling molecule in pathways that control the cytoskeleton. Its activation is known to occur with mechanical stimulation of cell membranes [14] and to play a role in both the development of synapses as well as synaptic plasticity [15,16]. Moreover, RhoA is present in both rod and cone synaptic terminals [9,10]. Most recently, we reported that the Rho kinase (ROCK) inhibitor AR13503 reduces spherule retraction and improves scotopic full-field electroretinogram (ffERG) responses two days after iatrogenic detachment followed by spontaneous reattachment [17]. The improvement in scotopic responses was highly correlated with a reduction of synaptic retraction, indicating that cytoskeletal control after an injury can reduce cellular dysfunction. Thus, in addition to examining the synaptic structure, we have assessed the responses of cone cells in experiments that repeat the conditions previously used for rod cells by testing cone photopic ERG responses two days after detachment and spontaneous reattachment. We show that AR13503 can improve cone synaptic function even though the pattern of damage in cone pedicles is distinct from rod spherule damage. 

In these two-day experiments, iatrogenic retinal detachment, followed by spontaneous reattachment, follows a scenario similar to a surgical subretinal application or insertion of a viral vector or stem cell transplant [18,19]. ROCK inhibition, therefore, may be relevant for iatrogenic detachments created for these purposes. Moreover, the experiments were done on an adult pig, a diurnal animal whose eyes are similar to human eyes in many respects: the retina is similar in size, vascularization, and cellular structure, having both rod and cone cells and an area of high cone density, and the area centralis, similar in function to the human macula [20,21,22]. Porcine and human ERGs are similar as well [23,24]. Using porcine retinal detachment as a model of retina injury, therefore, enhances the translational significance of the results. Additionally, to assess whether ROCK inhibition can be applied not only at the time of detachment but also sometime after injury, we have tested whether treatment with ROCK inhibition can be delayed. 

Our results indicate that ROCK inhibition protects both rod and cone synapses from injury, and thus its application may improve recovery from retinal detachment and reduce the likelihood of unsatisfactory outcomes reported after surgical retinal reattachment procedures [25,26,27,28,29,30,31,32].

## 2. Materials and Methods

### 2.1. Animals

Three-month-old male or female Yorkshire pigs weighing 30 kg were obtained from Animal Biotech Industries (Danboro, PA, USA) and kept on a 12 h light/12 h dark cycle. Animals were housed in an Association for Accreditation and Assessment of Laboratory Animal Care (AAALAC)-accredited pathogen-free facility, one animal to a pen. They were subject to overnight fasting with access to water ad libitum before surgery. Experimental procedures and methods of euthanasia were approved by the New Jersey Medical School Institutional Animal Care and Use Committee (IACUC ID 202000121, Protocol PROTO999900995, 30 November 2020) and adhered to the ARVO Statement for the Use of Animals in Ophthalmic and Vision Research. A total of 19 animals and 38 eyes were used. Further descriptions of the animals can be found in Table A1 (Appendix A). 

### 2.2. Retinal Detachment and Experimental Design

For consistency, retinal detachments were always created between 9 a.m. and 12 p.m. in the morning. Animals were injected with atropine (0.02 mg/kg; VetUS, Henry Schein, Dublin, OH, USA) and sedated with an injection of ketamine (20 mg/kg; Mylan Institutional LLC, Galway, Ireland) or telazol (3–4 mg/kg; Zoetis, Parsippany-Troy Hills, NJ, USA) and xylazine (2.2 mg/kg; Lloyd Lab., Shenandoah, IA, USA), all administered intramuscularly. After 5–10 min, a peripheral venous catheter was inserted through the auricular vein, and the animal was intubated with an endotracheal tube. In some cases, to deepen anesthesia, propofol (3 mL; Zoetis, Parsippany-Troy Hills, NJ, USA) was administered. To maintain anesthesia, the animals were supplied with 0.5% to 3.0% isoflurane in oxygen using a ventilator. Lactated Ringer’s solution was infused intravenously at a rate of 8 mL/kg/h. Vital signs (oxygen saturation, heart rate, and body temperature) were monitored and maintained within the normal range throughout the experiment.

**Figure 1:**
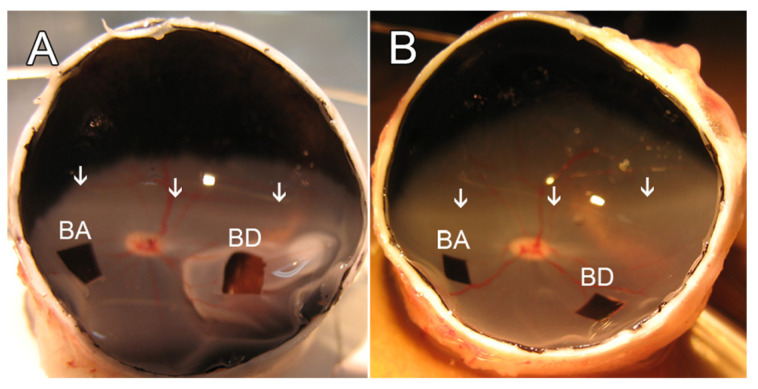
Pig eyes after enucleation and fixation. (**A**) A right eye 2 h after the formation of a detachment. The retinal detachment is present in the nasal inferior retina, below the area centralis (arrows). The boxes indicate the location of samples removed for sectioning. BD, sample of detached retina; BA, sample of attached retina. (**B**) A right eye two days after formation of a detachment. The detachment has spontaneously reattached. BD, sample of formerly detached retina; BA, sample of attached retina. Arrows indicate the area centralis, which extends from nasal to temporal retina.

For surgery, pupils were dilated with a topical application of 1% Tropicamide (Bausch & Lomb, Tampa, FL, USA) and 2.5% phenylephrine (Paragon Bioteck, Portland, OR, USA). A standard 3-port vitrectomy was performed using 20 g instrumentation. The posterior hyaloid was detached over the area centralis using active suction, and a core vitrectomy was completed. During and after vitrectomy, the vitreous cavity of the eye was perfused with a balanced salt solution (BSS; Alcon, Fort Worth, TX, USA) containing 2 μg/mL epinephrine (Henry Schein, Dublin, OH, USA). To mitigate variability in anesthesia levels or other possible biological variables, each animal served as its own control, with one eye receiving BSS and the fellow eye the ROCK inhibitor. A 33 g metal cannula was used to inject BSS subretinally to create a retinal detachment (~10–15 mm in diameter) in the inferior nasal quadrant below the area centralis (Figure 1). For subretinal drug administration, the Rho kinase inhibitor AR13503 (Aerie Pharmaceuticals, Durham, NC, USA) was dissolved in the BSS to make the detachment. Immediately after the procedure, the sclerotomies were closed with a 7-0 vicryl suture. For intravitreal administration of a drug, 150 μL of 10 or 15 μm AR13503 dissolved in BSS was injected with a 30-gauge needle into the vitreous cavity (entering ~3 mm posterior to the limbus) with the fellow eye receiving an equal volume injection of BSS alone. The volume of the vitreous cavity was estimated to be 3 mL, and the final intravitreal concentrations were estimated to be 0.5 and 0.75 μm, respectively.

After RDs were created, the animals survived for an additional 2 to 4 h or for two days. For the 2–4 h procedures, animals were kept under anesthesia after detachments were made and then euthanized with 7 mL intravenous Euthasol (Vibrac AH, Fort Worth, TX, USA) for enucleation. For the longer survivals, the conjunctiva was sutured (7-0 vicryl) after the sclerotomies were closed, 1.6 mg (0.4 mL) Dexamethasone (Fresenius Kabi, Lake Zurich, IL, USA) and 0.1 g (0.5 mL) Cefazolin (WG Critical Care, LLC, Paramus, NJ, USA) were injected subconjunctivally, and Tobradex ointment (Alcon, Fort Worth, TX, USA) was applied topically. Once the animals had recovered, they were maintained in their cage, with constant monitoring, for an additional two days. Animals were administered pre- and post-operative intramuscular injections of buprenorphine (0.01–0.05 mg/kg; Reckitt Benckiser HealthCare, Hull, England) and enrofloxacin (10 mg/kg; Bayer HealthCare, Shawnee, KS, USA). At the two-day time point, the animals were again anesthetized, using the previous protocol, for ERG recording and structural analysis by fundus photography and optical coherence tomography (OCT), as described below, before being euthanized with 7 mL intravenous Euthasol for enucleation.

### 2.3. Full-Field Flash Electroretinogram (ffERG), Fundus Photography, Optical Coherence Tomography (OCT)

The procedures for recording ffERGs, fundus photography, and OCT were done under general anesthesia, as described above. For all three procedures, pupils were dilated, and accommodation was relaxed with topical application of 1% Tropicamide and 2.5% phenylephrine hydrochloride drops. Adjustable lid specula were used to keep the eyelids separated. ERGs were recorded in animals that had two-day survivals both before retinal surgery and two days after surgery. Fundus photography and OCT were performed in the animals two days after retinal surgery to confirm the status of the retina. 

During electroretinography, flashes were produced, and responses were recorded using a UTAS ERG system with a BigShot Ganzfeld stimulator (LKC Technologies, Inc., Gaithersburg, MD, USA). The pig’s head was placed inside the ganzfeld bowl, and bilateral ERGs were recorded simultaneously using ERG-Jet electrodes (Fabrinal SA, La Chaux-de-Fonds, Switzerland) placed on the cornea. The cornea was kept moist with a hypromellose ophthalmic demulcent solution of 2.5% (Akorn, Inc., Lake Forest, IL, USA). The reference electrode was placed at the midline of the forehead, about the same distance from both eyes. The ground electrode was placed on the right shoulder of the animal. The stimulus protocol was based on the International Society for Clinical Electrophysiology of Vision (ISCEV) standard for clinical ffERG [33]. Briefly, to isolate the cone a- and b-waves, the animal was light-adapted for 10 min to a 20 cd/m^2^ background. The ffERG was recorded to strobe flash intensities of 3 cd.s/m^2^ (0 db) with an interstimulus interval (ISI) of 0.5 s (15 samples). A notch filter (60 Hz) and 85 Hz low pass filter were applied during data analysis using Matlab^®^ (The Mathworks, Natick, MA, USA) to eliminate noise. The a-wave amplitudes were measured from the beginning of the a-wave to the a-wave trough, and a-wave implicit times were measured from stimulus onset to a-wave peak. The b-wave amplitudes were measured from the a-wave trough to the b-wave peak, and b-wave implicit times were measured from stimulus onset to the b-wave peak. Data points were automatically identified, and values were calculated by custom-made script in Matlab^®^ (The Mathworks). Individual responses were analyzed, and aberrant waveforms were rejected before averaging.

### 2.4. Sample Preparation and Immunohistochemistry

After enucleation, a 5 mm slit was made at the ora serrata, and the eyes were immersed in 4% paraformaldehyde (EMS, Hatfield, PA, USA) for 15 min. The eyes were then opened, the anterior segment and any remaining vitreous humor were removed, and eyecups were fixed overnight at 4 °C. Samples were collected from areas of the retina that had been detached and from areas of the retina that had not been detached as illustrated in Figure 1. Retinas were immersed in 30% sucrose overnight at 4 °C. On the following day, specimens were embedded in OCT compound (Sakura Finetek, Torrance, CA, USA) at room temperature for 2 h, then frozen and cut into 15-μm-thick sections using a cryostat, as described previously [8]. 

Procedures for immunolabeling have been previously described [8]. Briefly, sections were washed two times with 0.3% Triton X-100 in PBS (PBS-T) and blocked with 10% blocking buffer (10% normal donkey serum, 33% PBS-T, and 57% PBS) for 1 h at room temperature. For visualization of cone synapses with confocal microscopy, sections were incubated in antibodies for CtBP2 (1:100, Fisher Scientific, Pittsburgh, PA, USA) and PSD-95 (1:100, Cell Signaling Technologies, Danvers, MA, USA) and peanut agglutinin (PNA) conjugated to FITC (1:100, Vector Labs, Burlingame, CA, USA) either overnight at room temperature or for 36 h at 4 °C. The following day the sections were washed three times with 0.3% Triton-100 in PBS and incubated with secondary antibodies conjugated to Alexa Fluor 546 or 647 (1:100, Life Technologies, Norwalk, CT, USA) and again with PNA-FITC for 90 min at room temperature or overnight at 4 °C. For STED microscopy, the primary antibody for CtBP2 was the same, but the secondary antibody was conjugated to STAR 635P (1:100, Abberior GmbH, Gottingen, Germany), whereas the PNA label used was conjugated to Alexa Fluor 594 (1:50, Life Technologies). In addition, incubation with the primary antibody and PNA was for 36 h, and with the secondary antibody and PNA, 24 h, at 4 °C. The resulting stronger labeling was useful for the increased intensity of the lasers needed to visualize the specimen with STED. For visualization of rod synapses with confocal microscopy, sections were incubated in an antibody to SV2 (1:100 dilution, Developmental Studies Hybridoma Bank, Iowa City, IA, USA) overnight at 4 °C. The next day, the sections were washed three times with 0.3% Triton-100 in PBS and incubated with a secondary antibody conjugated to Alexa Fluor 488 (1:100, Life Technologies) for 90 min at room temperature, followed by nuclear staining with 1 μg/mL propidium iodide (PI, 1:100, Sigma-Aldrich, St. Louis, MO, USA) or TO-PRO3 (1:500; Life Technologies) for 5 min at room temperature. After two washes with 0.3% Triton-100 in PBS, sections were covered with Fluoromount-G medium (Southern Biotech, Birmingham, AL, USA) or Diamond Antifade 5 (Fisher Scientific, Boston, MA, USA) and preserved under coverslips sealed with nail polish. For all immunohistochemistry, sections from retinal areas to be compared were placed on a single slide so that they were labeled together under the same conditions; control sections were also processed simultaneously with experimental sections but without primary antibodies.

### 2.5. Quantification of Cone Pedicles

All data were collected by a person masked to the sample identifications. Sections were examined using confocal microscopy (Nikon A1R+ HD Confocal Microscope, Melville, NY, USA) with a 60× oil immersion objective N.A. 1.4. Laser power and gain were set to obtain unsaturated images. In sections, locations for imaging were selected at random, and stacks of approximately 40 images of 0.20 μm optical thickness to allow for adequate overlap were created. Laser power, gain, and scanning rate were unchanged during the collection of images from a single experiment. Images were denoised before analysis. Enhancements in brightness and contrast were performed (Photoshop 7.0 software; Adobe, CA, USA) only for presentation purposes. 

Two samples (BSS-treated attached retina, BA; BSS-treated detached retina, BD; AR13503-treated attached retina, AA; AR13503 Detached retina, AD) from each eye, four samples per animal, were obtained (Figure 1); two to three stacks of images were taken of each retinal sample. Cone pedicles were determined by the PNA label, which, in the outer plexiform layer (OPL), only labels the base of cone synapses. Stacks were scanned to follow intact ribbons, i.e., where both the beginning and the end of the ribbon were apparent in the stack, and ribbon length was measured. About 12–50 ribbons in each sample were measured. The number of ribbons varied because, in some conditions, ribbons were lost. The measurements are reported in micrometers.

### 2.6. Quantification of Rod Synapses

As mentioned above, data were collected by a person masked to the sample identifications. Sections were examined using confocal microscopy (model LSM510; Carl Zeiss Microscopy, Jena, Germany or Nikon A1R+ HD Confocal Microscope) by scanning 1 μm optically thick sections with a 63× N.A. 1.4 or 60× oil immersion objective N.A. 1.4, respectively. Laser power and gain were set to obtain unsaturated images. Laser power, gain, and scanning rate were unchanged throughout a single experiment. Enhancements in brightness and contrast were performed (Photoshop 7.0 software) only for presentation purposes. 

As above, two samples (BA, BD or AA, AD) from each eye, four samples per animal, were obtained (Figure 1); 30–45 images were taken of each retina sample, and data were collected from 2 to 4 sections per sample, examining at least three different areas of each section. SV2 immunolabeling in the ONL was analyzed as described [8]. Briefly, a binary mask was created for each image, the ONL was outlined, and the labeled pixels in the ONL were counted using ImageJ software (version 1.45s; NIH). The measurements are reported as pixels per micrometer of ONL length.

### 2.7. Stimulated Emission Depletion (STED) Microscopy and 3D Imaging

Sections immunolabeled for STED microscopy were examined with a Stellaris 8 tau-STED Super Resolution Microscope (Leica Microsystems, Wetzlar, Germany) using a 93× glycerol-immersion objective lens N.A. 1.3, Leica’s white laser for excitation, and a 755 nm laser for depletion. Two to three stacks of 0.14 μm optical sections were collected from each sample, with approximately 160 images per stack.

Stacks were viewed with Imaris software (v 10.0; Imaris for Core Facilities, Oxford Instruments, Tubney Woods, UK). CtBP2 immunolabel, for ribbons, and PNA label, indicative of the base of the cone pedicle, were viewed as 3D images by surface rendering of the fluorescent images. In some cases, all remaining fluorescent labels and CtBP2 immunolabels not associated with the PNA marker, diagnostic for rod ribbons, were removed to increase the clarity of the cone pedicles and associated ribbons for presentation. 

Analysis of ribbons per pedicle was made from the rendered CtBP2 label; the surface area of the base of the pedicles was measured from en face views of the rendered PNA-labeled base of the pedicles using NIH ImageJ 1.5t (https://imagej.nih.gov/ij/index.html accessed on 3 January 2023). The perimeters of basal PNA labeling were outlined using the freehand selection tool, and area measurements were performed on ellipses fitted to PNA perimeters.

### 2.8. Statistical Analysis

For statistical analyses, the negative-binomial model with random effects, a generalized estimating equation (GEE) [34], or a linear model with random intercept was applied. Eyes within animals were randomized for BSS or AR13503 treatment. GEE and random intercept models were applied to estimate the parameters of a linear regression model while adjusting for the intrinsic correlation between repeated outcomes on the same subject, e.g., eyes within animals, multiple areas within eyes, and so on. 

Statistical analysis was performed with SAS (version 9.4). The graphics were produced using GraphPad Prism 5.1 and Matlab^®^ (The Mathworks, Natick, MA, USA). Box plots created with GraphPad show the median as a bar and the mean as a plus sign; the whiskers represent 1.5X the interquartile range (IQR) with dots as outliers. Figure 13 was done with Matlab. The whiskers represent the standard deviation, the bar is the mean, and the dots represent all data. We set alpha (type I error rate) at 0.05. Reported *p*-values were obtained via GEE analysis or the linear model unless otherwise noted.

## 3. Results

### 3.1. Normal Cone Synapses

**Figure 2:**
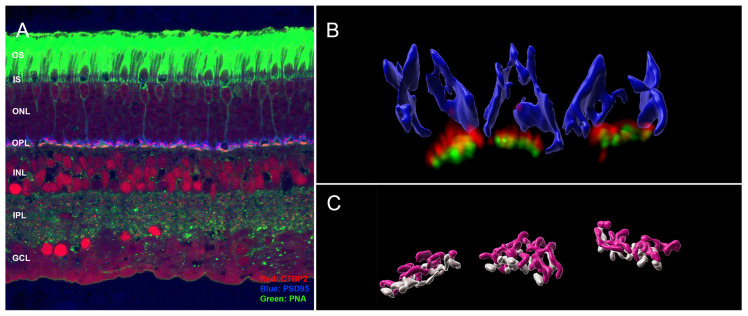
Normal porcine retina. (**A**) Low magnification of the retinal layers. CtPB2 (red) labels the ribbons in the outer plexiform layer (OPL) and is prominent in some nuclei. PSD-95 (blue) labels all photoreceptor synapses in the OPL. PNA (green) labels cone cells. It is present at the outer (OS) and inner (IS) segments, the cone axon, and the base of the pedicles. PNA is also present in the inner plexiform layer (IPL). ONL, outer nuclear layer; INL, inner nuclear layer; GCL, retinal ganglion cell layer. (**B**) High magnification of cone pedicles as seen from a stack of images taken with conventional confocal microscopy. PSD-95 has been surfaced rendered and labels the inside surface of the pedicles. PNA appears as discreet spots, and ribbons (CtBP2) as elongated spots. (**C**) Cone pedicles as seen from a stack of images taken with STED microscopy. The ribbon (pink) and PNA (gray) labels have been surface rendered. STED microscopy with surface rendering shows additional detail in the ribbon structure and PNA aggregates.

Cone synapses were examined from normal, unoperated animals with confocal and STED microscopy to allow for comparison with cone pedicles after a detachment injury with or without treatment with the ROCK inhibitor AR13503. All images were from paraformaldehyde-fixed and frozen tissue. Sections for conventional confocal microscopy were triple-labeled for (see graphic abstract and Figure 2): (1) CtBP2, a homolog of the B domain of the synaptic ribbon protein RIBEYE, present in rod and cone terminals [35], and identical to a transcription repressor leading to the label of retinal nuclei in the sections as well [36]; (2) PSD-95, a scaffolding protein that coats the inner surface of both the rod and cone presynaptic terminals [37,38] excluding the base of the synapses; and (3) PNA, a lectin which lines the membranes only of cone cells extracellularly and thus is a cone-specific marker [39]. PNA is bright along the cone’s inner and outer segments, lightly labels the cone cell body and axon, and then becomes bright again at the base of the cone pedicles; for STED microscopy, only CtBP2 and PNA were labeled.

**Figure 3:**
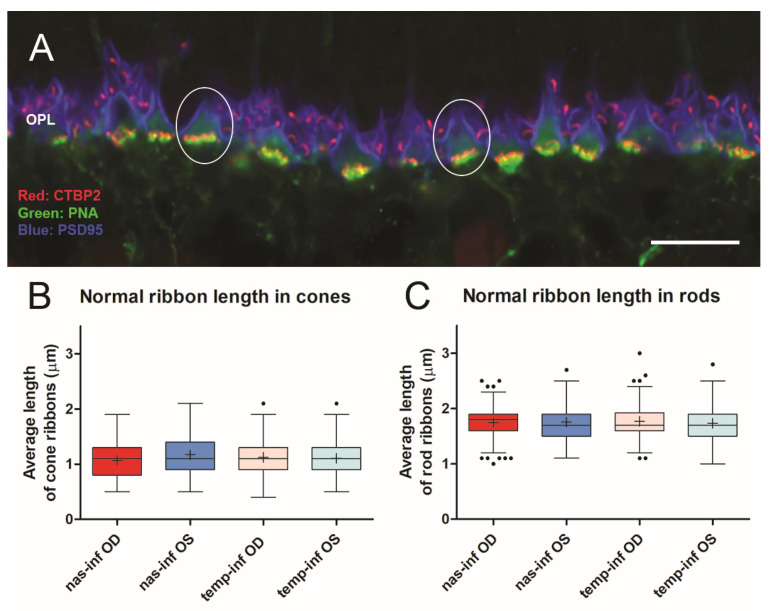
(**A**) Normal retina seen with conventional confocal microscopy. Cone pedicles (ovals) lie amongst rod spherules. Cone ribbons lie just above the PNA, whereas rod ribbons occupy a band about 5 μms wide and surround the pedicles. A stack of similar images was used to quantitate the length of photoreceptor ribbons. (**B**) The length of cone ribbons. The size of ribbons is relatively consistent across the inferior nasal and temporal retina. n = 2 animals. (**C**) The length of rod ribbons. Rod ribbons are longer than cone ribbons but also consistent in size across the inferior retina. n = 2 animals. OS, left eye, OD, right eye. For box plots, whiskers are 1.5X IQR; dots are outliers. Bar = 10 μm.

As in most mammalian retinas, the cone pedicles in the porcine retina form a narrow band in the outer plexiform layer (OPL) facing the bipolar dendritic processes (Figure 2). The rod spherules lie at multiple layers in the OPL distal to the cone synapses, and the rod ribbons, therefore, occupy a band about 5 μms wide. With conventional confocal microscopy, the PSD-95 label outlines the shape of the cone pedicle, which usually appears somewhat triangular (Figure 2 and Figure 3). CtBP2 does not label the entire height of the plate-such as an electron-dense ribbon structure that is seen in electron micrographs (EM), as it is only labeling one component of RIBEYE, which itself makes up only about 67% of the mature ribbon [40]. However, the label extends the entire length of the ribbon. The cone terminal has multiple ribbons in contrast to rod spherules, which have a single ribbon. Cone ribbons often appeared as a row of elongated points, just above (distal) a row of PNA dots which are presumably labeling the base of the pedicle between synaptic invaginations (Figure 2 and Figure 3). In conventional confocal microscopy, approximately 4–6 complete cone ribbons were seen in our image stacks of normal pedicles (Note: the number of complete ribbons visible in these stacks does not reflect the total number of ribbons present in a pedicle). Cone ribbons averaged 1.1 μms long in both the nasal inferior and temporal inferior quadrants that were analyzed (Figure 3). In some stacks, their horseshoe- or u-shape is apparent. Consistent with other mammalian retinas, the cone ribbons are shorter than rod ribbons; rod ribbons averaged 1.8 μms in length in nasal and temporal inferior quadrants (Figure 3).

**Figure 4:**
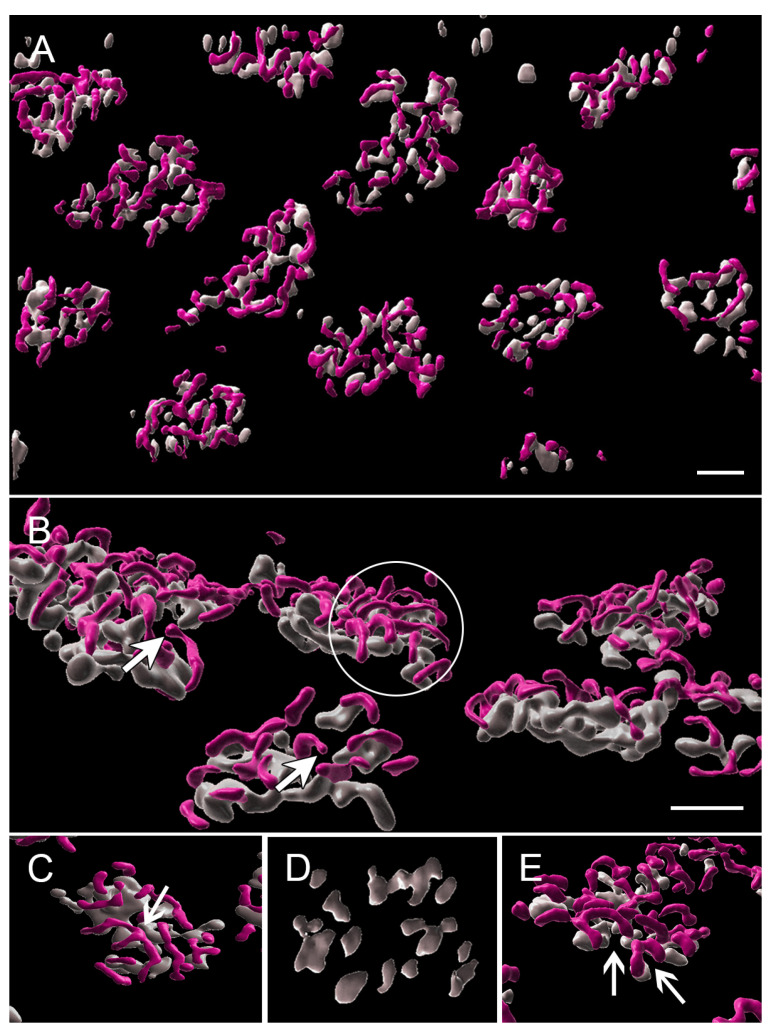
Normal retina from a stack of images taken with STED microscopy; fluorescent label was surface rendered. (**A**) The cone pedicles are regularly arrayed in this en face view. The PNA (gray) creates loose aggregates or islands of labels outlining the base of the pedicle. Ribbons (pink) appear as a loose network over the PNA label. (**B**) Most ribbons are single with a distinct horseshoe curvature (circle). Occasional swellings or knobs (arrows) appear along the ribbons. (**C**) Ribbons can also be in more complex branched configurations (arrow). (**D**) The aggregates of PNA are usually circular or oval. The spaces between the PNA are likely to be openings to synaptic invaginations. (**E**) Synaptic invaginations (arrows), defined by the curved ribbons, are bordered by a PNA label at the base of the pedicle. Bars = 2 μm.

With the 3D reconstruction of STED images and surface rendering of the fluorescent label, the PNA label for an individual pedicle appears as a loosely connected aggregation of islands of labels of various sizes (Figure 4). Cone ribbons are distributed uniformly above (distal to) the PNA label and generally are horseshoe- or u-shaped as in conventional confocal microscopy (Figure 4). The “u” shape is a result of the fact that the ribbons parallel the surface of the synaptic invagination where the processes of bipolar and horizontal cells lie. Additionally, in some cases, the ribbons may exhibit knobs or slight swellings at their ends (Figure 4). Finally, some ribbons have a y- or x-shape (Figure 4), as if there had been anastomoses of two or more ribbons. This finding has been previously illustrated in serial block face scanning EM in the mouse retina [41]. 

The aggregates of the PNA label are usually oval in shape and vary in size from 11.2 to 33.8 μm^2^ with an average area of 24.1 μm^2^ (n = 43 pedicles from two animals). We suggest that the “breaks” in the PNA label indicate the openings of synaptic invaginations. There is some difference of opinion about whether PNA is within the invagination or not. Unlike a previous report on the primate retina [42], we have never seen co-localization of PNA and Goalpha in the porcine retina (Townes-Anderson, personal observations). Instead, in STED images, we observe PNA on either end of horseshoe-shaped ribbons (Figure 4E), indicating its presence at the base where OFF bipolar processes contact the pedicle. Thus, our images support the report in mice by Sherry et al. [43]. 

A 3D reconstruction of STED images allows assessment of the total number of ribbons per pedicle. There were from 4 to 17 ribbons per pedicle, depending on the size of the PNA-labeled synaptic base, with an average of 12.3 ribbons per pedicle (n = 21 pedicles from two animals).

### 3.2. Intravitreal Administration of AR13503 Decreased the Loss of Cone Ribbons in 2-h Detachments

We have previously reported on the effects of detachment to the rod spherule. We discovered that rod spherules retract rapidly toward their cell bodies as soon as 2 h after detachment [8], so we initially examined cone synapses at a similar 2 h point after detachment. Additionally, as before, we tested the effects of ROCK inhibition on injury-induced synaptic changes. The ROCK inhibitor used in these experiments was AR13503, a ROCK inhibitor that we have shown to be more efficacious in reducing rod photoreceptor damage after detachment than other ROCK inhibitors [17]. AR13503 is the active metabolite of the FDA-approved netarsudil (AR13324) developed by Aerie Pharmaceuticals Inc. (Durham, NC, USA); the sustained release implant form is currently in clinical trials for diabetic retinopathy and age-related macular degeneration (phase 1, https://clinicaltrials.gov, (accessed on 1 May 2022)). AR13503 has a Ki of 0.2 nM for both ROCK 1 and 2 and Ki’s of 1 nM and 27 nM for PKA and PKC, respectively [44]. AR13503 reduces the amount of rod spherule retraction induced by retinal detachment both by subretinal injection during the creation of a detachment or intravitreal injection following detachment creation [17]. Here, we examined sections from experiments in which the drug was applied intravitreally immediately following detachment. Some specimens had been previously examined for rod spherule retraction, and some came from newly performed experiments following the exact same protocols. 

We created retinal detachments in both eyes of a single animal and injected one eye with the ROCK inhibitor diluted in BSS (to reach 0.75 μm AR13503 in the vitreous) and the fellow eye with BSS alone. (Note: although previous studies showed 0.5 μm AR13503 gave the best outcome for protection against rod retraction [17], we had had some success with 0.75 μm AR13503 as well and had material that could be used for investigation of cone synapses from these experiments.) Once created, detachments remained for 2 h before euthanasia and enucleation. The presence of retinal detachment was confirmed after fixation and bisection of the eyes. Specimens for microscopic examination came from the center of the detachment and attached regions of the retina approximately 1.5 cm further temporally. Both locations were inferior to the area centralis, the region of high cone cell density (Figure 1). The pig is a dichromat. The S and M cones are found throughout the retina, with S cones constituting about 13% of the cones. The nasal and temporal inferior porcine retina, from which we have taken our samples, have a similar distribution of cone cell types [45]. The distribution of S cones in the porcine retina has no consistent topographical pattern and can vary from the existence of small clusters of S cones to small areas lacking S cones [45]. Although S cones are reported to be more sensitive to retinal detachment in some species (human, primate, and rat [46,47,48]), we have not attempted to distinguish the two cone cell types, and thus our description and analyses related to all cones collectively.

**Figure 5:**
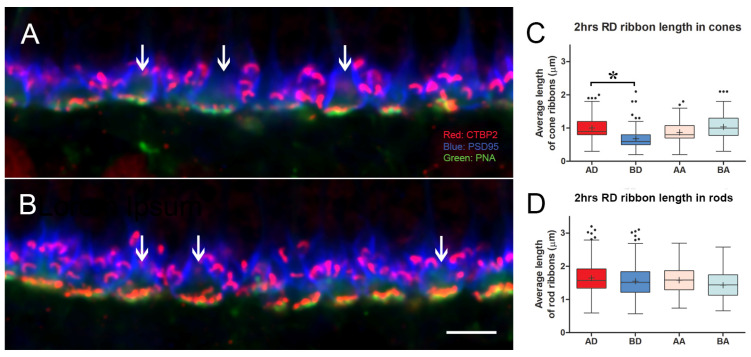
2 h after detachment. (**A**) Detached retina, untreated. Although ribbons are present in most pedicles, the arrows indicate pedicles with reduced and shortened ribbons. Rod ribbons are present between the pedicles. (**B**) Detached retina treated with AR13503. All pedicles have ribbons (arrows). (**C**) Length of cone ribbons decreased significantly in the untreated retina (BD) compared to the treated retina (AD), * *p* < 0.0001. There was no significant difference in ribbon length in attached retinas whether treated or not (BA vs. AA). n = 3 animals. (**D**) Length of rod ribbons showed no differences in the treated vs. untreated eyes in detached and attached retinas. n = 3 animals. For box plots, whiskers are 1.5X IQR; dots are outliers. Bar = 5 μm.

In conventional confocal microscopy of triple-labeled sections, cone pedicles looked relatively normal (Figure 5). The cone ribbons formed a single layer facing the bipolar dendrites. However, some decrease in the number of ribbons was apparent in the detached retina from a cursory examination. Quantitating the length of cone ribbons demonstrated that the untreated, detached retina had significantly shorter ribbons than the detached retina that had been treated with AR13503 by about 40% (BD vs. AD, *p* < 0.0001; Figure 5). The number of ribbons observed in stacks of conventional confocal images was also reduced in the untreated detached retina compared to the treated, detached retina (e.g., 3.0 vs. 4.8 ribbons/pedicle in 1 animal). The ribbons in the attached retina from untreated and treated eyes were similarly examined since we had previously observed disruption of the rod synapse in the attached retina [8]. However, there were no significant differences in the length of cone ribbons in untreated and treated retina (BA vs. AA Figure 5) in areas temporal to the retinal detachment. 

**Figure 6:**
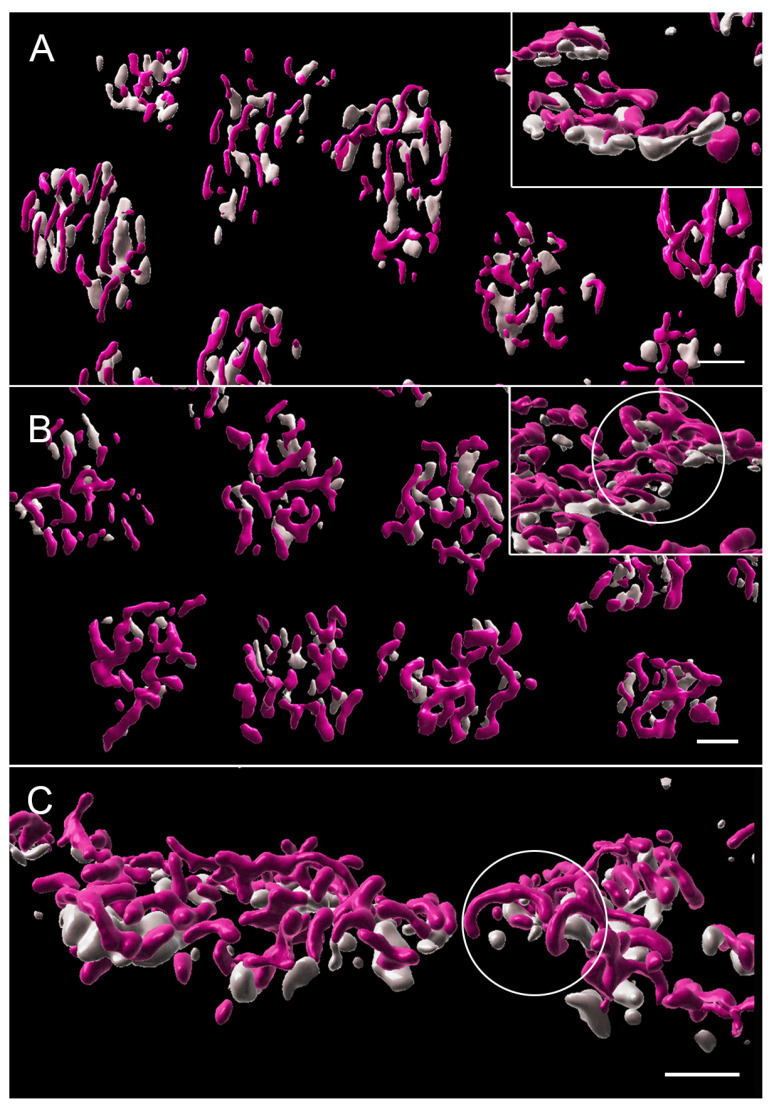
2 h after detachment. (**A**) Detached retina, untreated. The array of pedicles, highlighted by PNA label (gray), is less organized than the normal retina. The network of ribbons (pink) also looks less dense, indicating a loss of ribbons. Branched ribbons were uncommon. In many cases, the ribbons have lost their horseshoe curvature (inset). (**B**) Detached retina, treated. The array of pedicles looks more normal, as does the network of ribbons overlying the PNA aggregates. In many cases, the ribbons have the characteristic U-shape curvature (inset, circle). (**C**) Attached retina, untreated. The ribbons looked densely distributed over the PNA label, and the horseshoe shape was consistently present (circle). STED microscopy with surface rendering. Bars = 2 μm.

**Figure 7:**
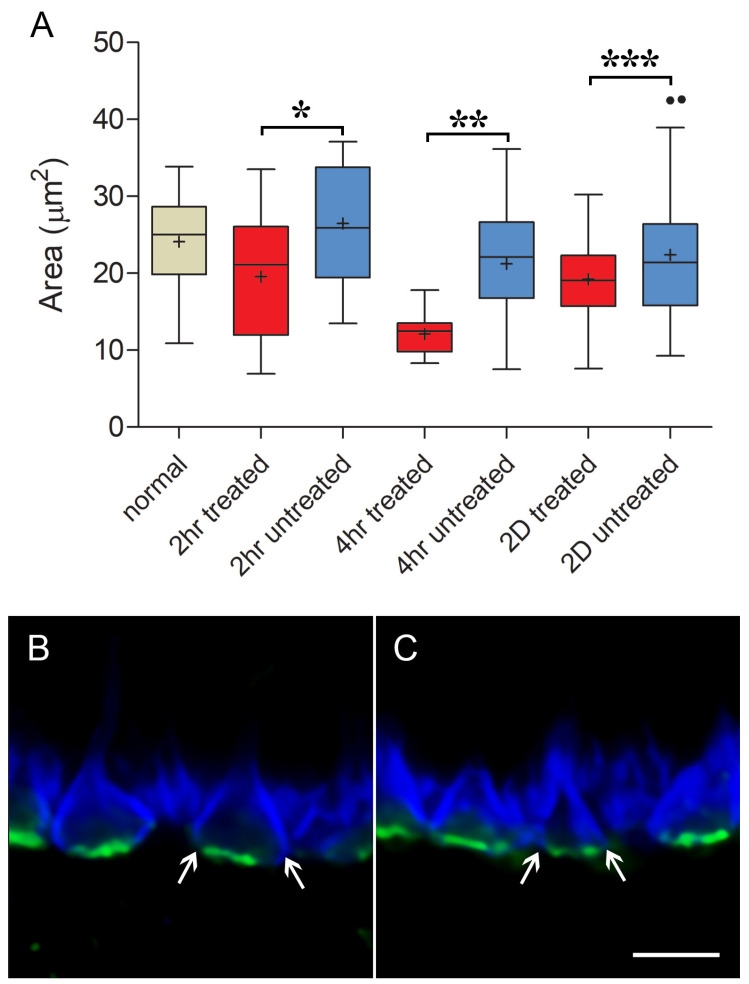
Size and shape of pedicles. (**A**) Area of pedicle base, measured from PNA label (see methods), at different times after detachment (2 and 4 h) and two days after detachment and reattachment (2D). Pedicle bases from 2 h, 4 h, and two-day treated retina are significantly smaller than those in untreated retina. For 2 h n = 41 pedicles from 1 animal, * *p* = 0.0004; for 4 h n = 28 pedicles from 1 animal ** *p*= 0.007; for 2D n = 72 pedicles from 2 animals, *** *p* = 0.049. Pedicles from 4 h treated (*p* < 0.001) and untreated retina (*p* = 0.042) and 2 h (*p* = 0.003) and 2D treated retina (*p* = 0.0028) are smaller than normal; for normal n = 42 pedicles from 2 animals. For box plots, whiskers are 1.5X IQR; dots are outliers. (**B**) 2 h after detachment. Detached retina, untreated. Pedicles look slightly larger and rounder than in normal retinas. PSD-95 (blue); PNA (green). The PNA label indicates that the presynaptic terminals are from cone cells. (**C**) 2 h after detachment. Detached retina, treated. The cone pedicles look slightly smaller and triangular in shape, and their sides are straighter than the untreated retina. Arrows point to the edges of the PSD-95 label. Bar = 5 μm.

With STED imaging and Imaris rendering, some pedicles of untreated retina appeared to be sparsely covered with ribbons. Cone ribbons also looked reduced in length with STED microscopy as quantitatively described above; the anastomoses of ribbons seen in the normal retina were less frequent, and the arched shape of the ribbons appeared more shallow (Figure 6). In a normal retina, the ribbon follows the curvature of the synaptic invagination; thus, the flattening of the ribbons strongly suggests there was some flattening of the invagination. Flattening of synaptic invaginations in cone pedicles has been previously described in detached retinas, but the data come only from detachments of three days duration or longer [4]. In contrast, in the treated, detached retina, most ribbons were normally arched, although some loss of the characteristic horseshoe shape in the treated retina could be found (Figure 6). Finally, the bases of the pedicles were significantly smaller in area, as determined by the PNA label, in the treated retina compared to the untreated retina (negative-binomial model, *p* = 0.0004, Figure 7) and compared to normal (*p* = 0.003). Immunohistochemical expression of PNA is relatively stable in detached rat retina compared to S cone opsin [46]; in the detached feline retina, the PNA label does not decrease for several days and remains at the synaptic terminal even after the labeling in the interphotoreceptor matrix has disappeared [7]. Changes in measurements based on the PNA label, therefore, are unlikely to be due to changes in PNA expression.

Thus, detachment results in the shortening, flattening, and disappearance of cone ribbons. ROCK inhibition applied at the time of detachment reduces these injury-induced changes. Unexpectedly, treated retinas had pedicles with a smaller presynaptic base. Both rounding and flattening of the shape of the feline pedicle have been described in longer-term detachments in vivo [4,49]. In vitro, we have seen rounding of porcine cone pedicles after 2 h of culture and spreading and flattening after 24 h [10]. It is possible that untreated pedicles were beginning to round up and lose their invaginations, whereas those treated with the ROCK inhibitor were not and were held in a more elongated configuration leading to a smaller base as well as better retention of the synaptic invaginations. Examination of the PSD-95 label confirmed that differences in pedicle shape existed between the untreated and treated retina (Figure 7). 

For rod cells, although rod spherules retract into the outer nuclear layer after retinal detachment, there is no change in length from rod ribbon length in the normal retina (Figure 3). In addition, an analysis of the total CtBP2-labeled pixels in the rod zone of the OPL of untreated versus treated detached retina showed no difference (data not shown), suggesting there is no loss of ribbons. Thus, rod ribbons appear unaffected by detachment or ROCK inhibition.

Since some of the specimens had previously been examined for rod spherule retraction (2 h data in [17]), we can conclude that (1) rod spherule retraction and cone ribbon shortening occur together in the detached, untreated retina and (2) both occur quickly, within hours of detachment. However, in the attached retina temporal to the detachment, where rod spherule retraction is present, albeit to a lesser degree than in the detached retina [8], significant cone ribbon shortening or loss of ribbons was not seen. These structural changes in the cone synapses, therefore, did not spread to the attached retina.

### 3.3. Delayed Intravitreal Administration of AR13503 Decreased Injury-Induced Changes in Both Cone and Rod Synapses

We have reported that the activation of RhoA increases by over 1.5-fold 2 h after retinal detachment in vivo [8]. For in vitro preparations of pig retina, activation remained high for at least 24 h [8]; in vivo, RhoA-GTP levels are higher 24 h after detachment compared to the control retina, but this increase did not reach statistical significance (n = 4 animals, *t*-test, *p* = 0.07, unpublished data). These data suggest that RhoA activation dissipates with time but may be present long after an injury has occurred.

In order to determine if ROCK inhibition could produce protection for photoreceptor synapses not just if initiated immediately during an injury but also if initiated a short time after injury, we applied the drug 2 h after detachment. Retinal detachments were made in both eyes of animals by subretinal injection of BSS. After 2 h, AR13503 was injected intravitreally to reach a concentration of 0.5 μm. Fellow eyes received an intravitreal BSS injection. The pigs survived an additional 2 h before euthanasia and enucleation. Thus, the total time from the original injury was 4 h. 

**Figure 8:**
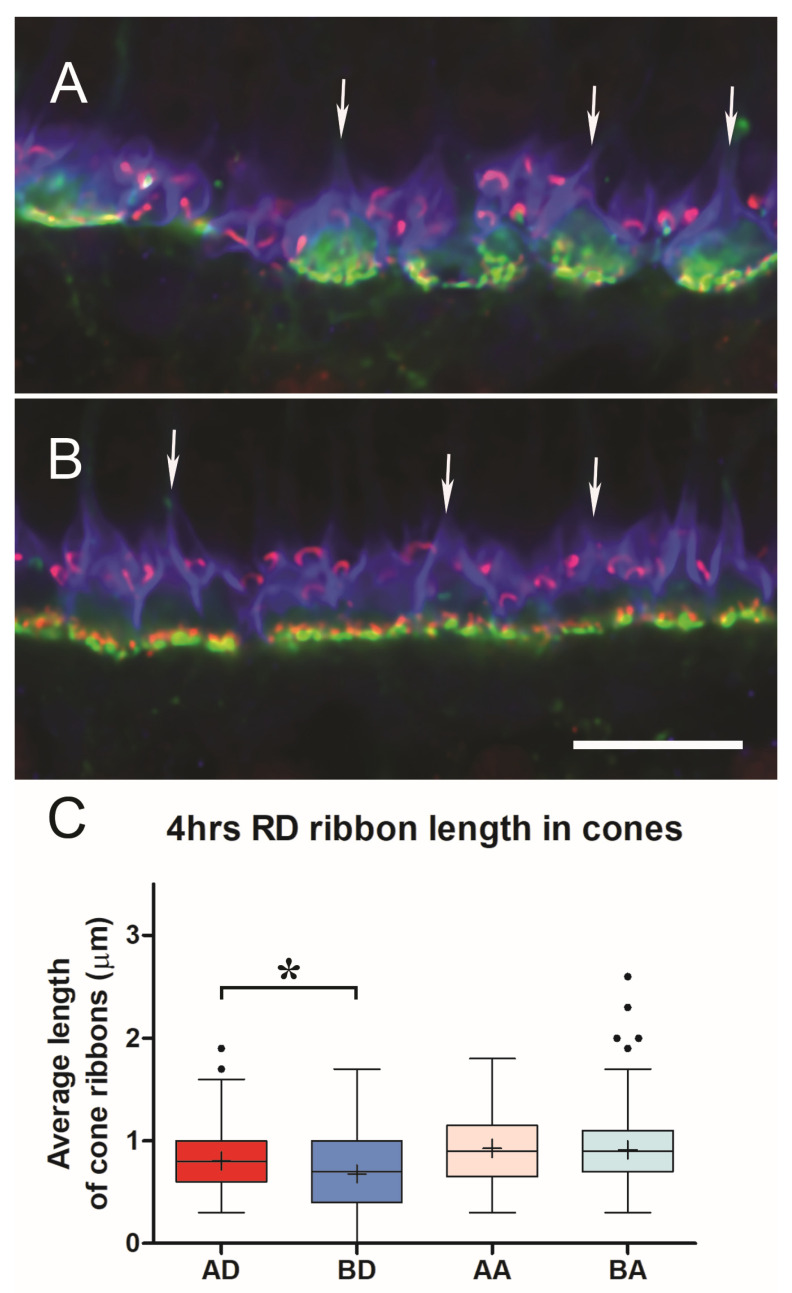
4 h after detachment. (**A**) Detached retina, untreated. Pedicles are rounded in shape (arrows), and cone ribbons are short. Rod ribbons are present above (distal to) the base of the pedicles. (**B**) Detached retina treated with AR13503 after a two-hour delay. Pedicles have the more usual triangular shape (arrows), and ribbons appear longer. (**C**) Length of cone ribbons in the untreated detached retina (BD) is significantly smaller than those in the treated retina (AD), * *p* < 0.05. Length of ribbons in attached, untreated, and treated retinas (BA vs. AA) showed no significant differences. n = 4 animals. For box plots, whiskers are 1.5X IQR; dots are outliers. Bar = 5 μm.

Cone ribbons in conventional confocal microscopy were shorter in the untreated detached retina compared to the treated detached retina (BD vs. AD, *p* < 0.05, Figure 8). Although the difference in the data between treated and untreated detached retinas was significant, the variability between animals was striking. In one animal, the difference between untreated and treated retina was negligible, whereas, in another animal, there were numerous empty pedicles (i.e., no ribbons) in the confocal stacks of the detached untreated retina compared to the treated eye. Variability between animals is not unexpected, as individuals would be expected to show differences. However, with detachment, there is also variability within the detached retina. We have observed differences between sections analyzed at different points in a single retinal sample and have therefore used multiple animals and multiple images for statistical analyses. Our observations are supported by others who report a high level of variability in the number of TUNEL-positive cells across detached rat retina [46] as well as in outer segment length, outer segment/retinal pigmented epithelial interface, mislocalization of rod opsin, and rod neuritic sprouting into the inner retina after reattachment of feline retinas [4,50,51]. In some cases, these variables contribute to a morphological “patchwork” appearance of photoreceptor recovery [50]. In the untreated detached retina, cone ribbons were shorter than in the normal retina (Mann–Whitney *p* = 0.009). In contrast, there were no apparent differences in cone ribbon length or number between the untreated and treated attached retina, and the cone ribbon lengths appeared normal (BA vs. AA, Figure 8). 

**Figure 9:**
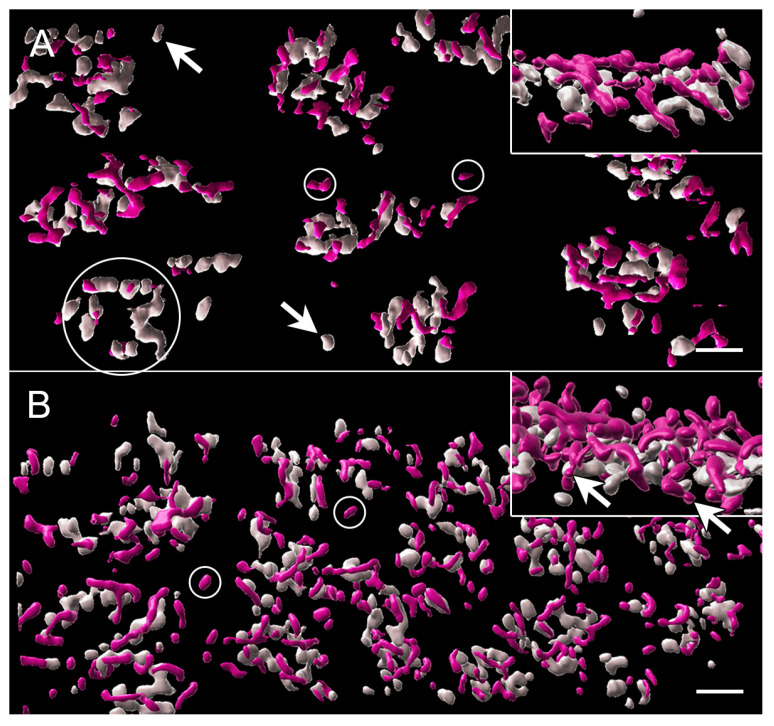
4 h after detachment. (**A**) Detached retina, untreated. PNA aggregates (gray) have reduced numbers of ribbons (pink), which appear generally shorter than normal and, in some cases, thinner. Some pedicle bases are nearly devoid of ribbons (circle). Isolated PNA labels (arrows) and ribbons (small circles) are present. Many ribbons are flattened against the pedicle base (inset). (**B**) Detached retina, treated. Pedicles appear more tightly arrayed than in the untreated retina. There are more ribbons associated with the PNA label but also some isolated ribbon fragments (circles). Some ribbons are arched, and many have “knobs” along their lengths and at their ends (inset arrows). STED microscopy with surface rendering. Bars = 2 μm.

With STED microscopy, the visualization of the morphological differences in untreated and treated detached retinas in the 2 h delay experiments was enhanced: without ROCK inhibition, there appeared to be fewer, shorter ribbons, and many were flattened (Figure 9). With ROCK inhibition, there were more ribbons, which appeared longer and frequently had some curvature (Figure 9). The general flattening at the base of the pedicle, therefore, was pronounced with a longer (4 h) detachment, whereas the loss of synaptic invaginations was reduced or reversed with ROCK inhibition. Additionally, there was a “knobbiness” to the ribbons from the treated retina; in other words, skinny ribbon segments alternating with slight swellings or protrusions (Figure 9). Although these swellings were occasionally seen in other specimens, including in the normal retina, as discussed, the treated retina in these delayed-treatment experiments showed the knobs more frequently. There were also occasional aggregates of ribbon labels that were unassociated with PNA-labeled terminals (Figure 9). Finally, the PNA-labeled base of the pedicles was smaller for both untreated and treated detached retina than normal retina (negative-binomial model, *p* < 0.05, details in legend for Figure 7), whereas in the treated retina, as at 2 h, the PNA-labeled base was significantly smaller than in the untreated retina (negative-binomial model, *p* = 0.007, Figure 7).

**Figure 10:**
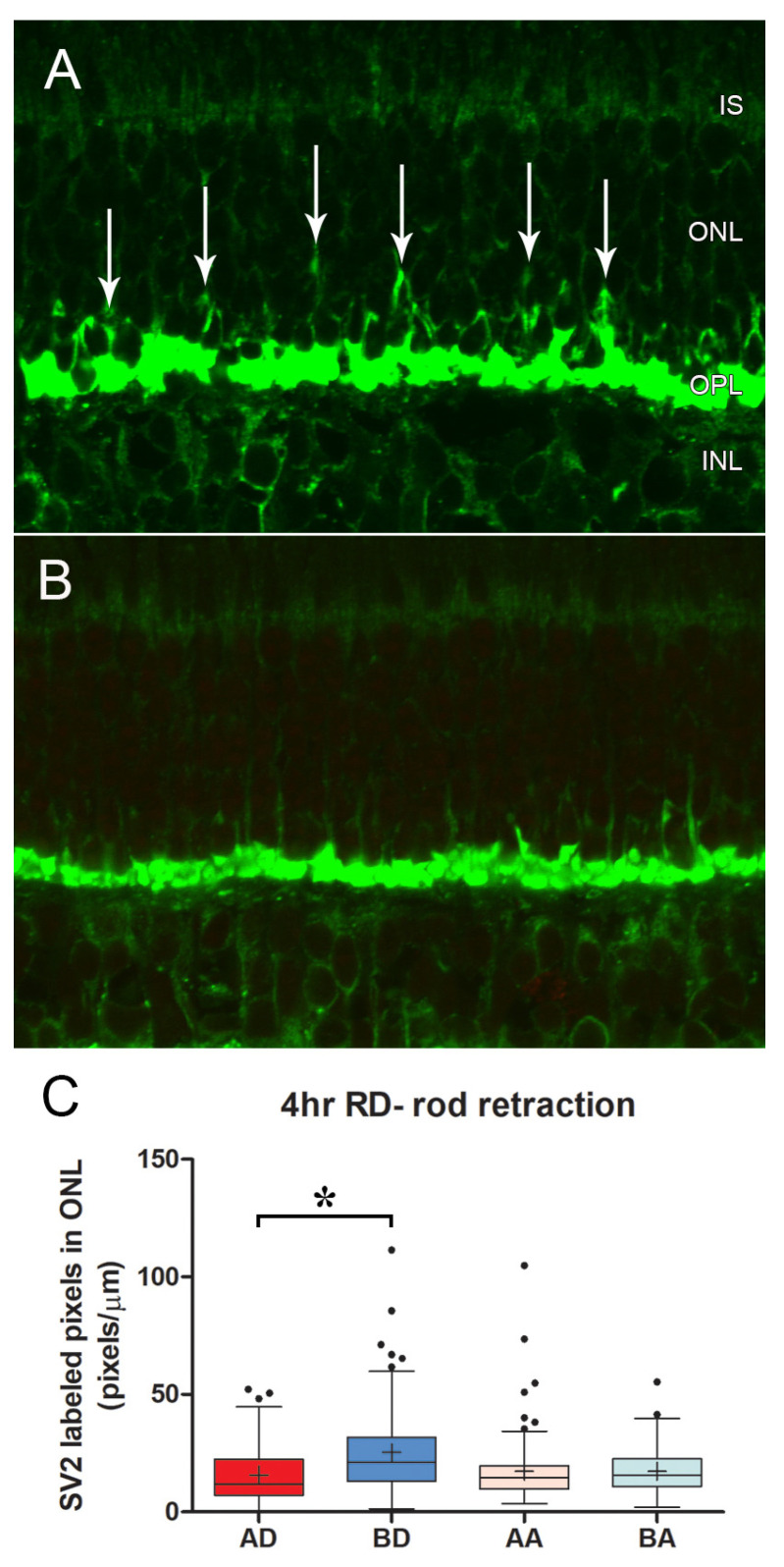
4 h after detachment. (**A**) Detached retina, untreated. Synaptic vesicles are labeled for SV2 (green). There are many retracted rod spherules (arrows). (**B**) Detached retina treated with AR13503 after a two-hour delay. Very few retracted rod spherules are present. (**C**) There is significantly more retraction of rod spherules in the untreated detached retina (BD) than in the treated detached retina (AD) * *p* = 0.03. There is no difference in the amount of retraction in attached, untreated (BA) vs. treated (AA) retinas. n = 4 animals. For box plots, whiskers are 1.5X IQR; dots are outliers.

Because ROCK inhibition seemed to be useful as a delayed treatment to prevent loss of ribbons in cone synapses, we also examined rod spherule retraction in the specimens from (1) some of the same animals or (2) animals subject to the same experimental conditions. The retraction was most severe in the untreated, detached retina but was reduced by 37.6% with ROCK inhibition (BD vs. AD, *p* = 0.03, Figure 10). As with the cone data, there was considerable variability; however, all four animals showed less retraction with drug treatment than without. There was no difference in retraction in untreated versus treated attached retina (BA vs. AA, Figure 10).

In a previous study with the ROCK inhibitor fasudil [11], we had not seen any reduction of rod retraction after a 2 h delayed treatment. Thus, AR13503 was more effective than fasudil in reducing synaptic injury to rod cells. Moreover, ROCK inhibition with AR13503 was effective in reducing injury for both cone and rod cells.

### 3.4. Structural and Functional Changes in Cone Synapses after Retinal Detachment and Spontaneous Retinal Reattachment

To assess the effects of reattachment on cone synaptic structure, we used a protocol that we had previously used for the examination of rod synapses [17]. Two days after detachment, most detachments have reattached spontaneously. Thus, we could examine cone synapses after reattachment as well as the effects of iatrogenic detachments followed by reattachment on cone function. 

For these experiments, ffERG baseline recordings were made before surgery, and then retinal detachments were made in both eyes. In one eye, the subretinal injection included 0.5 μm AR13503; in the fellow eye, the detachment was made with BSS only. After two days, ERG recordings were made again, the eyes were examined with fundus photography and OCT to determine if the retina had reattached, and the animal was then euthanized and enucleated. Only animals with reattachments were used in our analysis (Figure 1).

**Figure 11:**
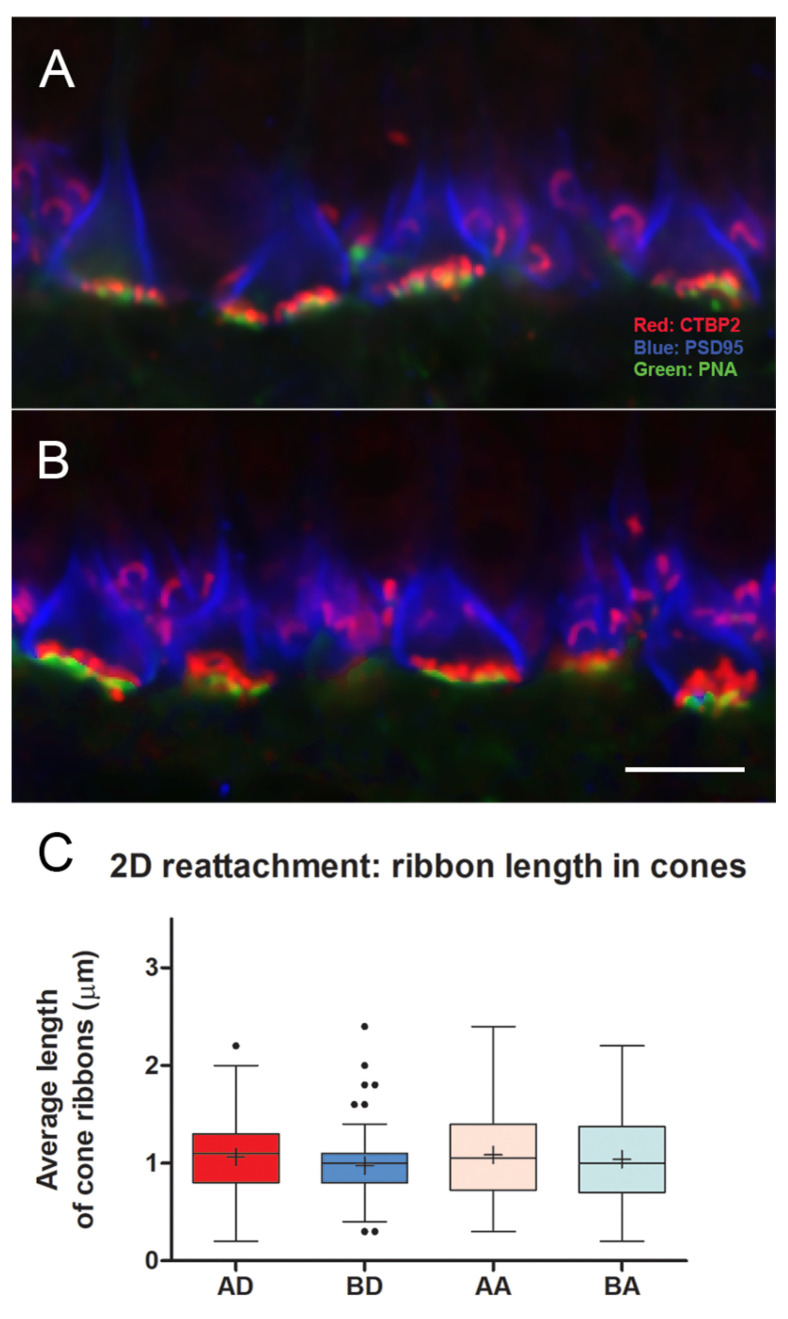
Two days after detachment and with reattachment. (**A**) Detached retina, untreated. (**B**) Detached retina treated with AR13503. Pedicles in both retinas show a normal distribution of ribbons. (**C**) Cone ribbons lengths showed no differences in all groups, AD, treated detached, AA, treated attached retina, BD, untreated detached, BA, untreated attached, n = 3 animals. For box plots, whiskers are 1.5X IQR; dots are outliers. Bar = 5 μm.

**Figure 12:**
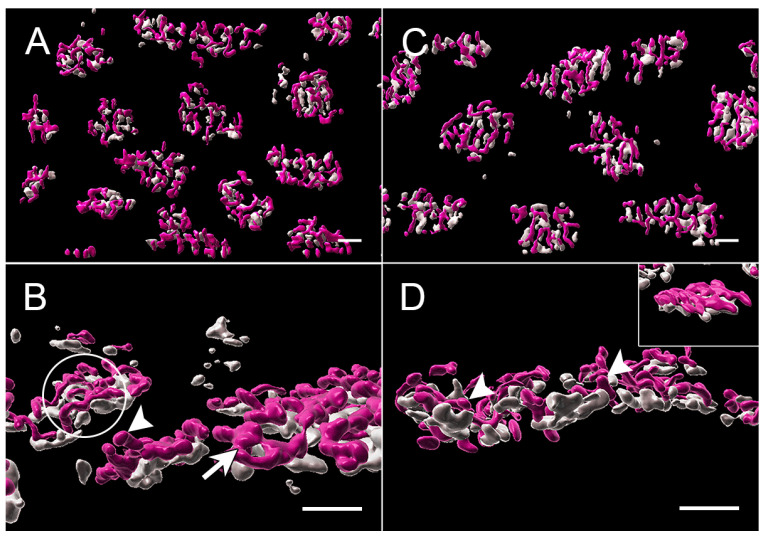
Two days after detachment and with reattachment. (**A**,**B**) Detached retina, untreated. The array of cone pedicles looks normal, and the density of the ribbon network also appears normal. However, there is considerable variability in the shapes of ribbons: ribbons with knobs (arrowhead) and ribbons with more complex shapes (arrow) are found on adjacent pedicles. Depths of ribbon arching are also variable, with some ribbons arched as in the normal retina and other ribbons shallower (circle). Some ribbons are completely flattened onto the pedicle base. (**C**,**D**) Detached retina treated with AR13503. The array of pedicles and density of the ribbons network is similar to the untreated retina. Many ribbons have knobs (arrowheads), similar to the untreated retinas; some ribbons appear flattened unto the pedicle base (inset). STED microscopy with surface rendering. Bars = 2 μm.

With conventional confocal microscopy, no differences in cone ribbon length were apparent between untreated vs. treated retina in the detached or attached retina (Figure 11). Ribbon lengths were consistent with those of normal retinas. With STED microscopy and Imaris rendering, the cone pedicles in untreated and treated retina looked similar after reattachment: Most PNA-labeled presynaptic bases were covered with multiple ribbons (Figure 12). In the untreated and treated retina, there were 11.9 (n = 14 pedicles from 2 animals) and 10.3 (n = 14 pedicles from 2 animals) ribbons per pedicle, respectively. Thus, the abundance of ribbons was comparable to the normal retinas, although occasionally, the number of ribbons was reduced. The complexity of the ribbons in terms of the number of anastomoses between ribbons was also similar to that in the normal retina. Notably, however, the curvature of the ribbons in both treated and untreated retina was reduced (Figure 12). Many ribbons appeared as “shallow” arches, unlike the typical u-shaped morphology seen in the normal retina. Some pedicles contained knobby ribbons as well. An additional abnormality was the size of the PNA-labeled base of the pedicles. Consistent with the 2 h and 4 h experiments, the treated retina had a PNA-labeled area that was slightly but significantly smaller than the untreated retina (negative-binomial model, *p* = 0.003) and normal (negative-binomial model, *p* = 0.049, Figure 7). Thus, even though cone ribbon length and number returned to normal after reattachment, synaptic invaginations, as detected by ribbon arching, were not as deep as in normal retinas. Some variability in the distribution of ribbons and a reduced area at the base of the pedicle also suggested that recovery at this time point was not complete throughout the areas of reattachment.

**Figure 13:**
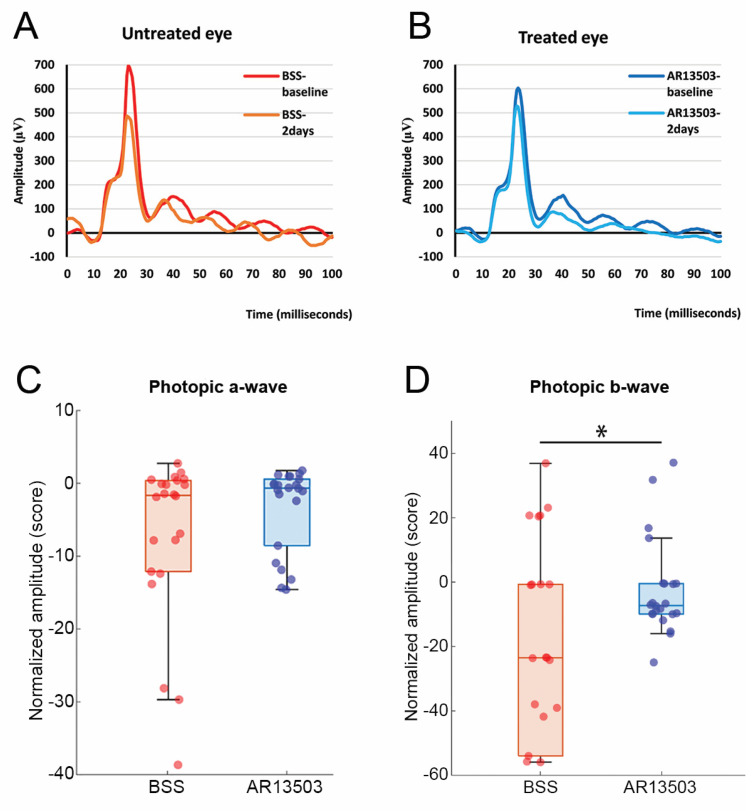
ERG recordings two days after detachment and with reattachment. (**A**,**B**) Representative waves of cone photopic b-wave for a BSS-untreated eye and an AR13503-treated eye. (**C**) Box plot showing normalized amplitude of photopic a-wave. There is no significant difference in a-wave amplitudes at two days from baseline recordings for both untreated and treated eyes. (**D**) Box plot showing normalized amplitude of photopic b-wave. BSS-untreated eyes have lower amplitudes at 2 days compared to baseline than AR13503 treated eyes, * *p* = 0.0004, n = 5 animals, an average of 5 recordings, repeated three times per animal. For box plots, whiskers are the standard deviation; dots represent all data points.

ERG recordings revealed that the function of the cone pedicles was not completely normal as well. Previously, to test for the synaptic function of rod spherules, we recorded and evaluated the scotopic b-wave [17]. We found that untreated retinas had lower amplitudes in their rod-specific, scotopic, b-wave. Here we examined the cone-specific, photopic b-wave using a similar analytical approach. To account for variability between animals, amplitudes were normalized as a percent of the baseline for each eye. We found that untreated retina had lower amplitudes than treated retina (*p* = 0.0004, Figure 13). The difference in the photopic b-wave was not due to transduction differences, as the a-waves were no different for untreated and treated retinas (Figure 13). The data indicate that treatment with AR13503 results in better synaptic transmission between cone photoreceptors and ON bipolar cells after reattachment.

### 3.5. Summary

We have now examined cone and rod presynaptic terminals 2–4 h after detachment and two days after detachment, followed by spontaneous reattachment. Both rod [17] and cone cells show a reduction in photoreceptor-bipolar transmission 2 days after a detachment, even though the retina has reattached. The similarity in functional injury is not paralleled by similar morphological patterns of injury. Indeed, the cone and rod photoreceptors show distinct structural changes after detachment. We have summarized our findings in a table to facilitate a comparison of these morphological distinctions (Table 1). Whereas reduced rod spherule retraction correlated with improved scotopic b-wave amplitude, no structural change in cone pedicles seemed correlated with the improved photopic b-wave associated with ROCK inhibition. Nonetheless, AR13503 reduced the morphological and functional changes caused by detachment in both cone and rod cells suggesting the potential usefulness of ROCK inhibition in the treatment of the retinal injury.

## 4. Discussion

Inhibition of activity in the RhoA pathway reduces synaptic damage to rod photoreceptors after injury, both in vitro and in vivo [8,9,10,11,12,13,17]. This current work is our first analysis of the effects of ROCK inhibition on cone synapses in vivo. We had, however, previously examined isolated cone cells from the tiger salamander and found that blocking the RhoA pathway with the ROCK inhibitor Y27632 stimulated neuritic process growth and formation of presynaptic varicosities in culture [9]. Since detachment in vivo does not produce neuritic sprouting in the time frame that we are examining, the in vitro work was not predictive of what we observed in vivo. However, we did observe in vitro that cone and rod salamander cells react differently to RhoA antagonists. Cone cells responded significantly faster and more robustly with the growth of processes and varicosities in the presence of Y27632, compared to rod cells [9]. One suggested conclusion was that RhoA activity levels and regulation are different in the two photoreceptor cell types. This suggestion is consistent with our current results, as cone and rod cells show quite different structural responses to detachment and, thus, presumably, to activation of RhoA signaling.

For cone cells in this porcine model of retinal detachment, there was a significant decrease in ribbon length, changes in ribbon morphology, and loss of ribbons within hours after detachment; changes in the length and number of ribbons were transient, being reversed with reattachment. In contrast, the ribbons in rod spherules seemed fairly stable, even though spherules retracted from their postsynaptic partners. They were clearly unaltered 2 h after detachment, the time point we examined quantitatively when cone pedicles have already shown some ribbon loss. In detached feline retinas, however, rod ribbons do eventually shorten after days of detachment in retracted and non-retracted spherules [4].

Under certain circumstances, both cone and rod ribbons are known to be plastic, changing shape from elongated plates, as seen with EM, to plate-like structures with swellings along the edges which serve to remove material from the ribbon to spherical electron-dense bodies that no longer appear as ribbons although they continue to be coated with attached synaptic vesicles. Spherical ribbons are usually detached from the active zone and found floating in the cytoplasm of the presynaptic terminal. These changes in size and shape have been reported to occur during the diurnal light phase, most notably in some strains of mice, with hibernation and a decrease in synaptic activity [52]. Moreover, the changes are reversible, with the greatest ribbon lengths achieved just after dark, along with increased visual sensitivity [53]. The knobbiness of porcine cone ribbons, as observed in 3D STED stacks of images, likely represents either (1) the process of reduction in ribbon size as spherical aggregates from elongated ribbons detach during periods of inactivity caused by detachment or (2) the reformation of ribbons after reattachment. In our specimens, the highest degree of knobbiness was seen in treated retinas, where the treatment had been delayed by 2 h. Additionally, small aggregates of ribbon labels were present unassociated with PNA-labeled synaptic bases. In the untreated fellow eyes, ribbon lengths were the shortest we observed. Treatment, therefore, may have halted the process of reduction mid-stream, leaving misshapen ribbons and free-floating ribbon spheres. Some knobs or protrusions are also present even after retinal reattachment.

Presynaptic calcium concentration appears to be a key determinant of ribbon size in photoreceptors. When photoreceptors are hyperpolarized in the light, calcium channels are closed, and the number of elongated plate-like ribbons is reduced [53,54,55,56]. Both EGTA and BAPTA, calcium chelators, likewise reduce the number of elongated ribbons and increase the number of spherical-shaped ribbon materials, whereas high levels of calcium reduce the number of spherical ribbons [56,57]. Interestingly, cone ribbons display higher sensitivity to calcium reduction than rod ribbons [57]. Most cone cell types have different L-type calcium channels at their terminals than rod cells [58], and cone terminals depend primarily on the Na^+^/Ca^+^ exchanger for maintaining calcium homeostasis, whereas rod synapses use primarily plasma membrane calcium ATPase (PMCA). These differences contribute to differing calcium dynamics in cone and rod synaptic terminals [59]. In the light, for instance, the estimated free [Ca^2+^] in cone pedicles is 3.2-fold lower than in rod spherules [59]. The mechanisms to lower calcium levels in cone pedicles with light-induced hyperpolarization may contribute to the increased sensitivity of cone ribbons in detachment-induced hyperpolarization compared to rod ribbons. Furthermore, cone ribbons, unlike rod ribbons, do not depend on piccolino, one of the molecular constituents of ribbons along with ribeye/CtBP2, to maintain an elongate shape [60]. Intraterminal free [Ca^2+^] may also contribute to the lack of ribbon changes in cone pedicles in the attached retina. 

Rod spherules retract after detachment in both the detached retina and throughout the retina in the areas of the attached retina [17]. Spherule retraction throughout the retina is consistent with increased RhoA activity in both detached and attached retina [8]. In contrast, cone terminal morphology seems normal in the attached portions of the retina after detachment. Cone cells in the attached retina would not be hyperpolarized as they are in the detached retina. In the dark, cones cells are known to have rapid neurotransmission, faster than synaptic kinetics at rod synapses. Thoreson [58] suggested that the differences in synaptic kinetics are due to differences in intraterminal calcium handling. Cone cells have higher calcium levels than rod cells in the dark [61], with particularly high calcium hots spots at the cone ribbon [62]. Thus, in the attached retina, where photoreceptors are generally in a depolarized state and not in prolonged detachment-induced hyperpolarization, high calcium in cone pedicles would favor the presence of mature elongate ribbons. 

Since the addition of a ROCK inhibitor reduced ribbon loss in cone pedicles after detachment, it is reasonable to suggest that there is a connection between RhoA activity and cone ribbon plasticity. Ribbons are not thought to have connections with the actin cytoskeleton; however, photoreceptor terminals contain myosin Va, an actin-based motor molecule linked to control of the size and shape of photoreceptor ribbons [63]. Cytoskeletal movement, in turn, is controlled by Rho signaling, but exactly how that results in changes in cone ribbons is currently unknown. Alternatively, the effect of ROCK on cone ribbons may come indirectly through interactions with voltage-gated calcium channels. ROCK interacts with smooth muscle L- and neuronal N-type calcium channels, causing reduced and increased calcium currents, respectively [64]. ROCK also interacts with T-type calcium channels. The kinase decreases currents in Ca_v_3.1 and 3.3 and increases calcium currents in Ca_v_3.2 channels [65]. Just recently, T-type calcium cells have been reported in mouse retinas at release sites exclusively in cone pedicles [66]. In the mouse, they are the Ca_v_3.2 type of channel. ROCK would increase calcium current if the cone pedicle regulation by ROCK were the same as dorsal root ganglion cells [65]. However, because of the highly variable effects of ROCK on voltage-gated calcium channels and the high specificity of regulation mechanisms for T-type channels [67], it is difficult to predict how ROCK inhibition may affect calcium current amplitude in pedicles. The connection between RhoA activity and ribbon plasticity warrants future investigation.

The normal arching of cone ribbons was observed to flatten after detachment. This flattening indicates the flattening or disappearance of synaptic invaginations. After detachment, the loss of invaginations seemed to occur simultaneously with changes in pedicle shape. Fisher and colleagues [4,49] reported that feline cone pedicles could either round up or flatten days after detachment. In vitro, we observed porcine pedicles to round up 2 h after detachment and then appear flattened and spread out by 24 h in vitro [10]. With EM, after several days of detachment, invaginations have completely disappeared [4]. These shape changes would necessarily involve the cytoskeleton, and it is therefore not surprising that loss of synaptic invaginations, as seen by flattening of ribbons, and rounding up, as seen with PSD-95 label, are reduced with ROCK inhibition. Similar to ribbon knobbiness, the depth of synaptic invaginations did not return to normal for many active zones after reattachment at two days. The lack of discernable differences between untreated and treated retina in arching of ribbons after detachment and spontaneous reattachment suggest there may be residual RhoA activity days after injury and that pedicle structural changes are no longer inhibited by the drug AR13503. In traumatic brain injury, activated RhoA is detected for months after injury [68]. Loss of synaptic invaginations could change the effectiveness or properties of synaptic transmission at the photoreceptor synapse since glutamate diffusion and, therefore, glutamate levels, as well as the local ionic environment in the synaptic cleft, may be affected [69,70] and the relationships of dendrites, with their postsynaptic receptors, to sites of glutamate release may alter [71]. The differences between the functional restoration of untreated and treated reattached retina may lie in part with subtle differences in the geometry of the synaptic invaginations, differences that we are not able to distinguish with current morphological techniques. 

In addition to the connection between the cytoskeletal control of presynaptic shape and synaptic invaginations and RhoA signaling, cytoskeleton and RhoA signaling also play a role in exo- and endocytosis at the synapse. ROCK signaling, for instance, reduced the size of the readily releasable pool of synaptic vesicles and increased the rate of synaptic vesicle endocytosis in central nervous system neurons [72,73]. Any imbalance in exo- and endocytosis adds to or removes plasmalemma at the active zone and can change synaptic efficacy. Reduction in endocytosis at photoreceptor synapses, for instance, slows transmission as calcium channels become more distant from vesicle release sites [70,74]. Cone synapses have a unique polyphosphoinositide phosphatase, Synaptojanin 1, which participates in clathrin-mediated endocytosis [75]. The presence of this unique protein indicates that the process of synaptic vesicle recycling differs in cone vs. rod cells. In addition, amphiphysin, also associated with clathrin-mediated endocytosis, is reduced in cones compared to rod terminals in mice [76]. It is possible that Rho-kinase-stimulated endocytosis may contribute to the rapid loss of synaptic invaginations in cones but not rod presynaptic terminals. Rod spherules do lose their invaginations in a feline model of retinal detachment but only slowly over a period of days [4]. Thus, Rho-kinase activity may alter cone presynaptic morphology and function after detachment directly by its effect on the cytoskeleton and indirectly through possible interaction with voltage-gated calcium channels and synaptic vesicle recycling. With reattachment, subtle differences in cone presynaptic structure and synaptic processes may still remain for the untreated and treated retina. Functional differences, however, could also be due to changes in molecular constituents postsynaptically. 

Congenital stationary blindness (CSNB) has an ERG phenotype in which the a-wave is normal, and the b-wave is missing. Multiple genes are known to cause this retinal disease. Many mouse mutants share a no-b-wave ERG phenotype. They are known collectively as *nob* mice. The mutated genes are expressed in the ribbon synapse either presynaptically, affecting glutamate release, or postsynaptically affecting the depolarization of ON bipolar cells. Significantly, when the problem is postsynaptic, the morphology of the ribbon synapse can appear normal both with light and electron microscopy [77]. After detachment and reattachment, we found that untreated and treated retinas did not appear different morphologically. There was a reduction in ribbon arching and the presence of knobs on ribbons in both untreated and treated retinas; otherwise, the synapses appeared normal. The abnormalities in ribbon arching and knobbiness observed with STED microscopy would most likely not be seen in light or conventional confocal microscopy or even in electron microscopy, where 3D reconstruction is rarely done and shows only a small portion of the active zone. So with conventional techniques, the synapses might be described as normal. Thus, we suggest that the functional problem, in the form of a lower photopic b-wave amplitude in the untreated porcine retina with a normal a-wave, may be due to some postsynaptic component of the synapses. In CSNB, mutations have been found in the metabotropic glutamate receptor (mGLUR6), the signaling components Goalpha and Gβ5, and in nyctalopin, an extracellular protein located at the base of the synapse. In cone synapses, nyctalopin appears distal to the ribbons, near the PNA label, and is necessary for synaptic transmission due to its association with the transient receptor potential melastatin channel, TRPM1 [78,79]. If this suggestion is correct, reduced cone b-wave amplitude would be due, at least in part, to reduced mGLUR6 signaling. Interestingly, in a report on the cone-specific ELFN2 synaptic adhesion protein, a binding partner of mGLUR6, a strong correlation was observed between reduced b-wave amplitude and reduced mGluR6 synaptic content [80]. Clearly, this hypothesis remains to be proven, but it would be consistent with the morphological and functional description of *CSNB1* and *nob* mice. For cone cells, the loss of postsynaptic signaling, however, would not be due to genetic abnormalities but rather to the injury response and would likely occur in a limited area. In contrast, for rod cells, we have shown a high correlation between increased rod axon retraction and reduced scotopic b-wave amplitudes [17]. Thus, the causes of reduced photoreceptor-bipolar transmission after retinal detachment are disparate for the two photoreceptor cell types. 

It is likely that every cell type in the eye contains RhoA and that ROCK inhibitors, when injected subretinally or intravitreally, could potentially influence activity in many cell types within the eye. For instance, the retinal vasculature responds to ROCK inhibition by relaxation [81]. Mechanosensitive TPRV4 channels on Müller cells cause increased RhoA activity resulting in increased GFAP expression and release of MCP-1, which, in turn, can cause photoreceptor apoptosis in retinal detachment [82]. ROCK inhibition can reduce glial reactivity [83]. Additionally, RPE structure and barrier function is restored in diabetic retinopathy with ROCK inhibition [81]. So AR13503 injections may have multiple effects intraocularly. Nonetheless, damage to the first synapse in the visual system may be the most negative feature of the retinal injury response to detachment and protection of this synapse, both its presynaptic and postsynaptic components, the most important therapeutic role for treatment with ROCK inhibitors. We have suggested, based on observation of rod synapses, that ROCK inhibitors could be included in iatrogenic procedures for gene and stem cell therapies where subretinal injections are used [17]. The additional information on cone synapses presented here reinforces this suggestion. In this report, we also demonstrate that damage to both cone and rod synapses is reduced even when the treatment is administered 2 h after retinal detachment. This response indicates that post-injury injections may also be useful for retinal trauma. Moreover, it may be beneficial to include a ROCK inhibitor during surgical reattachment as a mechanical perturbation, which can stimulate RhoA activity, is involved. Indeed, as we have previously mentioned (see Section 1), anatomically successful surgical reattachment in patients does not guarantee a return to normal vision. Functional and structural consequences of RhoA activity likely contribute to remaining visual losses. Our own and others’ work has shown, in humans [84] and animal models [4,17,51], that abnormal neuronal networks remain after reattachment, whereas rod neuritic sprouting only appears with retinal reattachment [4]. Application of a ROCK inhibitor during or after retinal injury may improve patient outcomes by reducing synaptic abnormalities. 

## Figures and Tables

**Table 1:** Synaptic Structural Changes in Untreated Detached Retina.

Cone Pedicles			
	2 h after Detachment	4 h after Detachment	2 days after Detachment and with Reattachment
Reduction in ribbon			
Length	xx	xx	o
Number	x	xx	o
Curvature	x	xx	x
Reduction in area of			
PNA-label	o	x	o
**Rod Spherules**			
Retraction of			
terminal	xx *	xx	x *

x or xx—relative difference from normal; o—no different than normal; * Data from [17].

## Data Availability

Please contact the corresponding author for information regarding data.

## Appendix A

**Table A1:** All animals used for this study. Although only pigs with dark, pigmented irises were accepted in this study, to minimize the possibility of depigmentation in the posterior segment, some areas of depigmentation were present in the fundus, as noted. (RD: retinal detachment; os: oculus sinister; od: oculus dexter; ou: oculus uterque) * Rod data from these animals on spherule retraction were used for a previous study [17]. ** Rod data from these animals on spherule retraction and rod ERGs were used for a previous study [17].

Animal ID	Sex	Notes	Depigmentation
** Control (terminal)**			
DOD6	F	Data not used because enucleation was several hours after euthanasia	
DOD16	M	Cone and rod data	
DOD21	M	Cone and rod data	
** Intravitreal 0.75 μm, 2 h RD (terminal)**			
69	F	Cone and rod data *	depigmentation in od
70	F	Cone and rod data *	
DOD14	M	Cone and rod data	
** Intravitreal 0.5 μm, 4 h RD (terminal)**			
DOD2	M	Cone and rod data	depigmentation in ou
DOD3	M	Rod data	
DOD4	M	Rod data	
DOD8	M	Cone data	
DOD12	M	Cone and rod data	depigmentation in os
DOD20	M	Cone data	
** Subretinal 0.5 μm, 2 Days (survival)**			
45B	F	Cone ERG data **	depigmentation in ou
48	F	Cone ERG data **	intraretinal hemorrhage in od(BSS)
49	F	Cone ERG data **	
50	F	Cone ERG data **	depigmentation in ou
74	F	Cone morphological and ERG data **	depigmentation in ou
DOD17	M	Cone morphological data	
DOD18	M	Cone morphological data	depigmentation in ou
